# Short Term Pre-Operative Oral Corticosteroids—Tissue Remodeling in Chronic Rhinosinusitis with Nasal Polyps

**DOI:** 10.3390/jcm10153346

**Published:** 2021-07-29

**Authors:** Kamil Radajewski, Paulina Kalińczak-Górna, Marek Zdrenka, Paulina Antosik, Małgorzata Wierzchowska, Dariusz Grzanka, Paweł Burduk

**Affiliations:** 1 Department of Otolaryngology, Laryngological Oncology and Maxillofacial Surgery, University Hospital No. 2, 85-168 Bydgoszcz, Poland; 2 Department of Otolaryngology, Phoniatrics and Audiology, Collegium Medicum, Nicolaus Copernicus University, 85-168 Bydgoszcz, Poland; paulinakalinczak@gmail.com (P.K.-G.); wierzchowskam@op.pl (M.W.); pburduk@wp.pl (P.B.); 3 Chair and Department of Clinical Pathomorphology, Collegium Medicum, Nicolaus Copernicus University, 85-009 Bydgoszcz, Poland; marek.zdrenka@cm.umk.pl (M.Z.); paulina.antosik@cm.umk.pl (P.A.); d_grzanka@cm.umk.pl (D.G.)

**Keywords:** tissue remodeling, oral corticosteroids, chronic rhinosinusitis

## Abstract

Chronic rhinosinusitis is a process involving a number of adverse changes in the mucosa of the paranasal sinuses and nasal polyps. The main histological features of tissue remodeling are changes in epithelial structure, oedema, degradation of ECM (extracellular matrix), angiogenesis, and subepithelial fibrosis. In this study, patients were divided into two groups: group 1—patients with CRSwNP (chronic rhinosinusitis with nasal polyps) taking a nasal steroid and an oral steroid in the preoperative period, and group 2—patients with CRSwNP taking only the nasal steroid in the preoperative period. All samples were subject to histopatologic evaluation. The aim of this study was to investigate the effect of oral corticosteroids and topical steroids on the tissue of paranasal sinuses. We have shown statistically significant decreases in tissue eosinophilia per 5HPF and decreased fibrosis in group 1. No significant differences were presented in the percentage of total tissue oedema, epithelium, neutrophils, basement membrane thickening and vessels. Using systemic administration of 40 mg of prednisone for seven days decreased the counts of eosinophils and decreased fibrosis in the nasal polyps tissue in CRSwNP.

## 1. Introduction

The European Position Paper on Rhinosinusitis (EPOS) 2020 defined chronic rhinosinusitis as an inflammation of the nose and paranasal sinuses characterized by two or more symptoms, one of which should be either nasal blockage/obstruction/congestion or nasal discharge (anterior/posterior nasal drip): ±facial pain/pressure ± reduction or loss of sense of smell. The symptoms must last for ≥12 weeks. There are two main types of chronic rhinosinusitis—with and without nasal polyps (CRSwNP and CRSsNP) [1].

The prevalence of CRS in different regions of the world ranges from 4.5% to 12% [2]; CRSwNP is often associated with other diseases: allergic rhinitis, asthma, gastropharyngeal reflux disease (GERD), sleep apnea and nonsteroidal anti-inflammatory drug (NSAID) [3,4]. Nasal polyps were defined as edematous, semitranslucent masses originating from the mucosal linings of the sinuses and prolapsing into the nasal cavities [5]. Grayson et al. proposed defining CRS depending on the extent of inflammatory changes in the paranasal sinuses—when the inflammatory changes are located bilaterally or occupy more than the functional and anatomical compartment in the paranasal sinuses, we refer to a diffuse (bilateral) disease, while in a case where the inflammatory changes are unilateral or are limited to the functional and anatomical compartment, we are talking about a limited disease (however, in this case, oncological vigilance should be maintained in order to implement urgent diagnostics and treatment) [1,6]. Th2 immune response is often involved in CRSwNP by increasing the production of IL-4, IL-5 and IL-13, leading to the expression and activation of mast cells, basophils, eosinophils, and goblet cell hyperplasia [7]. The study aimed to investigate the effect of oral steroids (OCS) and topical steroids on tissue remodeling in nasal polyps. There are very few similar studies in the literature, so further research is needed to investigate the effects of oral steroid therapy on the tissue remodeling in nasal polyp.

## 2. Materials and Methods

The prospective controlled study included patients diagnosed with CRSwNP who were qualified for functional endoscopic sinus surgery (FESS).The patients had not been operated on before. The patients were divided into two groups: group 1 (*n* = 42)—patients with CRSwNP taking a topical steroid in the preoperative period (at least 4 weeks before the procedure) and an oral steroid (40 mg per day of prednisone 7 days before the procedure). Group 2 (*n* = 23) included patients with CRSwNP taking only the topical steroid in the preoperative period, at least 4 weeks before the procedure. After FESS procedure, the dose of the oral steroid was gradually reduced by 10 mg every 5 days.

Inclusion criteria were adult patients (≥18 years), diagnosed CRS based on EPOS criteria, and qualified for FESS. Exclusion criteria were age < 18, diabetes, and suspicion of a malignant process in the nasal sinuses. In both studied groups, the percentage distribution of the occurrence of bronchial asthma and allergies was similar; therefore, both groups were considered homogeneous in terms of comorbidities that may have an impact on the obtained results.

Nasal polyps were removed from nasal middle meatus during FESS—the tissue was macroscopically inspected and fixed in 10% buffered formalin at pH = 7.2 for further evaluation. Formalin fixed, paraffin embedded (FFPE) tissue block was cut and stained with hematoxylin-eosin (H and E). Selected paraffin block was cut into 5 µm thick sections, using a manual rotary microtome (Accu-Cut, Sakura Finetek, Torrance, CA, USA) and then placed on slides. Hematoxylin and eosin (H&E) staining was used to evaluate the cellular content and general histoarchitecture of tissue specimens.

After tissue preparation, the slides were evaluated for histological examination: tissue eosinophilia, epithelium (mild, moderate, severe), neutrophil infiltrate (present or absent), basement membrane thickening (BMT) (present or absent), edema (absent or present), fibrosis (no fibrosis, mild, moderate) and vessels. Eosinophil counts were reported as an average count of eosinophils per five high power fields (HPF), and, similarly to other authors, dichotomized to ≤10 versus >10 eosinophils per HPF. Following EPOS 2020, tissue eosinophilia was defined as >10 per HPF. As a result of wide range of blood vessels size and distribution within stroma, it was decided to evaluate their mean count in 20 HPF.

A statistical analysis of the size of the test sample was carried out in order to determine the significance at the level of *p*-value < 0.05 using the R software (ver. 4.0.3) (Boston, MA, USA). A value of *p* < 0.05 was considered as statistically significant. Statistical methods were used for the evaluation—Chi-squared test and Fisher’s exact test. The study protocol was approved by the ethics committee of our institution (KB 635/2016). This examination was conducted in accordance with the Helsinki Declaration.

## 3. Results

Our study project analyzed 65 patients with CRSwNP, including 42 patients treated with oral and topical steroid, and 23 treated only with topical steroid. There was no difference in age between groups: group 1—47.6 years—and group 2—47.1 years. Eosinophils counted in 5 HPF was decreased in group 1—20.9 ± 23.56 (mean ± standard deviation—SD)—compared to group 2—34.26 ± 21.78. There was no difference counted in vessels in 20 HPF between group 1—11.37 ± 3.77—and group 2—11.79 ± 2.75 (Table 1).

Observed between group 1 and 2 were statistically significant decreases in tissue eosinophilia per 5HPF (57.14% group 1, 82.1% group 2; *p* = 0.038) and fibrosis (no fibrosis—57.14% group 1, 52.17% group 2; mild—30.95% group 1, 47.83% group 2, moderate—11.90% group 1, 0% group 2; *p* = 0.014). No significant differences were present in the percentage of total tissues oedema (*p* = 0.49), epithelium (*p* = 0.96), neutrophils (*p* = 0.42), basement membrane thickening (*p* = 0.725) and vessels (*p* = 0.725) (Table 2).

Patients were also divided into age groups and three groups were distinguished (Table 3): first group (22–39 years old), second group (40–59 years old) and third group (60–82 years old). In all age groups, statistical differences were demonstrated, especially in oedema (group 1—*p* = 0.001; group 2—*p* = 0.0264) and fibrosis (group 1—*p* = 0.002; group 2—*p* = 0.007). Tissue eosinophilia showed correlation in all age groups taking oral steroids treatment in relation to the group without oral steroids treatment (*p* = 0.048 to *p* = 0.07). A similar relationship was demonstrated in the vessels count (group 1—*p* = 0.041, group 2—*p* = 0.077). No significant differences was found in comparison of neutrophils (*p* = 0.077 and 0.113), epithelium (*p* = 0.346 and *p* = 0.074) and basement membrane thickening (*p* = 0.082 and *p* = 0.055).

## 4. Discussion

Tissue remodeling is a process that results in temporary or permanent changes in the histological composition of tissues. [8] The different patterns of CRS are characterized by different remodeling features. The main histological features of tissue remodeling in CRSwNP are hyperplasia, metaplasia in epithelial structure, oedema, reduced expression of TGFB1, degradation of extracellular matrix (ECM), angiogenesis and increased vascular permeability [9,10].

The treatment of chronic rhinosinusitis should be management with appropriate medical therapy (AMT) which includes: topical nasal steroid, saline rinses, educate techniques/compliance and consider use of oral corticosteroids. Usually, short term treatment with an OCS is a 7–21 day trial [1]. The use of short term OCS, according to numerous studies, showed reduction in the inflammatory response and a reduction in the size of nasal polyps (NP). Combination of an oral and topical steroid affects the hyposmia, the size of the polyps and the flow of air through the nose more effectively than using them separately. Moreover, that treatment modulates nasal polyp mucosa by epithelial repair, regulation of tissue remodeling markers, increased collagen content and reduced tissue eosinophilia [9,11,12]. Preoperative oral steroids use in FESS reduced blood loss, shortened operative time and improved surgical field quality [13].

The study demonstrates the effects of using oral and topical corticosteroids on changes in tissue remodeling in nasal polyps. In our study, we observed that oral corticosteroid treatment reduced tissue eosinophilia and fibrosis in nasal polyps. The reduction in tissue eosinophilia after systemic steroid therapy has also been demonstrated in many previous studies. Jankowski et al. reported that systemic steroids appear to be significantly more effective at reducing eosinophil infiltration than topical steroids in selected patients [14]. In addition, de Borja Callejas et al. showed that OCS modulates the remodeling of NP tissue by promoting epithelial repair, regulating tissue remodeling markers (metaloproteinases—MMP-1, MMP-2, MMP-7, MMP-9), increasing total collagen content, and reducing tissue eosinophil infiltration [9]. However, some studies do not show such a relationship with steroids (intranasal and systemic) or the leukotriene receptor antagonist used in the preoperative period with any histopathology marker [15].

The use of preoperative OCS may cause false negative results for tissue eosinophilia; however, the result may depend on the dose of the drug—Fujimoto et al. showed that oral administration of betamethasone in a dose of 0.5 mg for seven days before surgery did not decrease the number of eosinophils in the nasal polyps [16]. Brescia et al. performed a histopathological analysis of NP tissues assessing eosinophil counts and eosinophil aggregates (defined as >20 eosinophils/HPF)—an increased amount of both eosinophil counts and eosinophil aggregates were found; interestingly, patients with nonsteroidal anti-inflammatory drug exacerbated respiratory disease (NERD) had a significantly higher tissue eosinophil count than asthmatic and allergic patients [17]. Topical glucocorticosteroids have a strong anti-inflammatory effect and are the most common treatment for patients with CRSwNP, but this therapy is not sufficient for all patients [18]. The anti-inflammatory effect of glucocorticosteroids inhibits the transcription of pro-inflammatory mediators, which results in a reduction in the inflamed sinus mucosa, edema, nasal congestion, and cell infiltration, which facilitates the drainage and ventilation of the paranasal sinuses [5,19]. Corticosteroids can substantially suppress phases of the inflammatory process, e.g., they inhibit eosinophil cationic protein (ECP), IL-5 and IgE. They also reduce inflammation recruitment cells, fibroblast proliferation and extracellular synthesis matrix proteins [12]. Systemic steroids are indicated in the treatment in patients with partially or uncontrolled disease (VAS > 5, symptoms of CRS present on most days of the week) and its use is recommended for two treatments per year [1]. Qualification for the use of oral steroid therapy should take into account the patient’s comorbidities (including diabetes, arterial hypertension, gastric and duodenal ulcer disease, and psychiatric disorders) [20]. Otherwise, topical steroids has been proved to be safe and well tolerated, with an incidence of adverse events no greater than those observed with a placebo [19]. In our study, we showed that oral steroid therapy affects the number of eosinophils in the polyps tissue, but does not affect angiogenesis—a similar number of vessels was shown in both groups.

Basement membrane thickening (BMT) has been associated with increased duration of CRS symptoms with coincidence of asthma. This might suggest a better response to a faster implementation of more aggressive treatment of CRS [21,22]. BMT is also positively correlated with the CT score and tissue eosinophilia in CRS [23]. It was observed that tissue eosinophilia was increased in the group of the older patients, which may be related to the duration of the inflammatory process in the sinuses of the nose, but these results require further studies and analyzes. Oral steroids promote epithelial repair in NP via the upregulation of the AP-1 (especially c-Jun oncoprotein) network and its related genes (COX-2, IL-6, AREG, HBEGF and EGR1) [24].

In our study, we showed a reduction in the severity of tissue fibrosis in individual age groups, however, this may be related to the CRS endotype—fibrosis is less common in CRSwNP than in CRSsNP. However, further research is needed in this case because of the possible increase in collagen deposition in the ECM following oral steroid therapy [9]. Tissue response to the administered oral steroid therapy may depend on the patient age—in the older age group there was no significant decrease in the severity of tissue oedema compared to younger age groups. Some authors report that the processes observed in CRS as repair and remodeling can occur in healthy people in response to airborne contaminants, airborne infectious agents, and other environmental challenges [25]. Nasal mucosa hyperreactivity (NHR) is related to the environment (humidity, temperature fluctuations, cigarette smoke) and to physical activity. The coexistence of NHR with CRS remains uncertain, however, Doulaptsi et al. demonstrated the presence of NHR in almost 2/3 of patients with CRS on the basis of a questionnaire survey. Due to the lack of a simple (standardized) diagnostic tool, it is currently difficult to assess the pathophysiological mechanism of NHR on CRS [26]. In the absence of an adequate therapeutic effect of CRS according to EPOS guidelines, allergic rhinitis and asthma, according to Allergic Rhinitis and its Impact of Asthma guidelines (ARIA), can be termed severe chronic upper airway disease (SCUAD). The interaction between chronic inflammation in the upper and lower respiratory tract may pose difficulties in treatment and disease control. The SCUAD concept could be helpful in designing future clinical and molecular trials—this could provide a better understanding of disease processes and determine a better treatment strategy for the patient [27,28]. Due to different clinical images and histotypes in CRS, the histopathological and immunohistochemical evaluation allows to determine the cytotype and CRS biomarkers. Endotyping allows to supplying the patient with rational treatment with greater success [29].

## 5. Conclusions

We observed that systemic administration of 40 mg of prednisone for seven days decreased the number of eosinophils and decreased fibrosis in the nasal polyps tissue in CRSwNP patients. The use of oral steroid therapy does not significantly effect other components of tissue remodeling. It seems that there is a need for further research on drug influence on tissue remodeling in chronic rhinosinusitis.

## Figures and Tables

**Table 1:** Patient characteristics.

	Group 1 (*n* = 42)	Group 2 (*n* = 23)
	Mean ± SD	Mean ± SD
Age	47.67 ± 13.31	47.13 ±11.94
Eosinophils (per 5 HPF)	20.9 ± 23.56	34.26 ± 21.78
Vessels (per 20 HPF)	11.37 ± 3.77	11.79 ± 2.75

SD—standard deviation; HPF—high power field.

**Table 2:** Histopatologic features from patients with chronic rhinosinustis with nasal polyps.

Variable	Group 1	Group 2	*p*-Value
N (%)	42 (64.5)	23 (36.5)	
oedema			0.486
no	13 (31.0)	4 (17.4)	
mild	19 (45.2)	12 (52.2)	
moderate	10 (23.8)	7 (30.4)	
Fibrosis			0.014
no	24 (57.1)	12 (52.2)	
mild	13 (31.0)	11 (47.8)	
moderate	5 (11.9)	0	
epithelium			0.096
mild	31 (71.8)	19 (82.6)	
moderate	9 (21.4)	4 (17.4)	
severe	2 (4.8)	0	
neutrophils			0.420
present	33 (78.6)	16 (69.6)	
absent	9 (21.4)	7 (30.4)	
eosinopfils (per 5HPF)			0.038
<10	18 (42.9)	4 (17.4)	
>10	24 (57.1)	19 (82.6)	
Basement membrane thickening			0.725
absent	20 (47.6)	12 (52.2)	
present	22 (52.4)	11 (47.8)	
Vessels (per 20HPF)			0.725
<11.5	20 (47.6)	12 (52.2)	
>11.5	22 (52.4)	11 (47.8)	

HPF—high power field.

**Table 3:** Tissue remodeling in particular age groups.

Variables	Group 1				Group 2			
Age Group	1	2	3	*p*	1	2	3	*p*
N (%)	42				23			
oedema				0.001				0.0264
No	5 (38.5)	8(38.1)	0		2 (25.0)	1 (9.1)	1 (25.0)	
mild	6 (46.1)	8 (38.1)	5 (62.5)		4 (50.0)	6 (54.5)	2 (50.0)	
moderate	2 (15.4)	5 (23.8)	3 (37.5)		2 (25.0)	4 (36.34)	1 (25.0)	
Fibrosis				0.002				0.007
no	4 (30.8)	16 (76.2)	4 (50.0)		5 (62.5)	3 (27.3)	4 (100)	
mild	8 (61.5)	3 (14.3)	2 (25.0)		3 (37.5)	8 (72.7)	0	
moderate	1 (7.7)	2 (9.5)	2 (25.0)		-	-	-	
epithelium				0.346				0.074
mild	9 (69.2)	16 (76.2)	6 (75.0)		8 (100)	8 (72.7)	3 (75.0)	
moderate	3 (23.1)	4 (19.0)	2 (25.0)		0	3 (27.2)	1 (25.0)	
severe	1 (7.7)	1 (4.8)	0					
neutrophil				0.077				0.113
absent	9 (69.2)	17 (81.0)	7 (87.5)		6 (75.0)	8 (72.7)	2 (50.0)	
present	4 (30.8)	4 (19.0)	1 (12.5)		2 (25.0)	3 (27.2)	2 (50.0)	
Eosinophils (per 5HPF)				0.048				0.070
<10	6 (46.1)	10 (47.6)	2 (25.0)		3 (37.5)	1 (9.1)	0	
>10	7 (53.9)	11 (52.4)	6 (75.0)		5 (62.5)	10 (91.0)	4 (100)	
Basement membrane thickening				0.082				0.055
absent	6 (46.1)	10 (47.6)	4 (50,0)		5 (62.5)	4 (36.4)	3 (75.0)	
present	7 (53.9)	11 (52.4)	4 (50.0)		3 (37.5)	7 (63.6)	1 (25.0)	
Vessels per 20HPF				0.041				0.077
<11.5	5 (38.5)	12 (57.1)	3 (37.5)		3 (37.5)	6 (54.6)	3 (75.0)	
>11.5	8 (61.5)	9 (42.9)	5 (62.5)		5 (62.5)	5 (45.4)	1(25.0)	

HPF—high power field.

## Data Availability

No public database has been created. All data are available from the authors of the work.
